# Exploring oxidative stress pathways in *Geobacter sulfurreducens*: the redox network between MacA peroxidase and triheme periplasmic cytochromes

**DOI:** 10.3389/fmicb.2023.1253114

**Published:** 2023-10-04

**Authors:** Pilar C. Portela, Leonor Morgado, Marta A. Silva, Lukas Denkhaus, Oliver Einsle, Carlos A. Salgueiro

**Affiliations:** ^1^Associate Laboratory i4HB – Institute for Health and Bioeconomy, NOVA School of Science and Technology, Universidade NOVA de Lisboa, Caparica, Portugal; ^2^UCIBIO – Applied Molecular Biosciences Unit, Department of Chemistry, NOVA School of Science and Technology, Universidade NOVA de Lisboa, Caparica, Portugal; ^3^Institut für Biochemie, Albert-Ludwigs-Universität, Freiburg, Germany

**Keywords:** *Geobacter*, oxidative stress, cytochrome, peroxidase, electron transfer, protein–protein interactions, NMR

## Abstract

The recent reclassification of the strict anaerobe *Geobacter sulfurreducens* bacterium as aerotolerant brought attention for oxidative stress protection pathways. Although the electron transfer pathways for oxygen detoxification are not well established, evidence was obtained for the formation of a redox complex between the periplasmic triheme cytochrome PpcA and the diheme cytochrome peroxidase MacA. In the latter, the reduction of the high-potential heme triggers a conformational change that displaces the axial histidine of the low-potential heme with peroxidase activity. More recently, a possible involvement of the triheme periplasmic cytochrome family (PpcA-E) in the protection from oxidative stress in *G. sulfurreducens* was suggested. To evaluate this hypothesis, we investigated the electron transfer reaction and the biomolecular interaction between each PpcA-E cytochrome and MacA. Using a newly developed method that relies on the different NMR spectral signatures of the heme proteins, we directly monitored the electron transfer reaction from reduced PpcA-E cytochromes to oxidized MacA. The results obtained showed a complete electron transfer from the cytochromes to the high-potential heme of MacA. This highlights PpcA-E cytochromes’ efficient role in providing the necessary reducing power to mitigate oxidative stress situations, hence contributing to a better knowledge of oxidative stress protection pathways in *G. sulfurreducens*.

## 1. Introduction

Bacteria from the genus *Geobacter* are a remarkable group of microorganisms capable of utilizing extracellular electron transfer mechanisms to harvest ATP (Lovley, 2011). For this, the microorganisms need to efficiently shuttle the electrons across the inner and outer membranes to reduce extracellular electron acceptors such as iron oxides or manganese oxides (Lovley, 2011). Contaminant compounds such as chromium, vanadium or uranium can also be used by this species to support cell growth (Ortiz-Bernad et al., 2004; Sanford et al., 2007; He et al., 2019). The seamless electron transfer (ET) is secured by a network of cytochromes that provide an effective interface between the inner and outer membranes (Reguera and Kashefi, 2019; Salgueiro et al., 2022). In fact, two distinct ET networks have been identified in *Geobacter sulfurreducens*: when the cell is exporting electrons to the exterior of the cell – current producing mode – or when it is receiving electrons from an external source – current consuming mode (Dumas et al., 2008; Kracke et al., 2015; Heidary et al., 2020). In the current producing mode, electrons originating from the oxidation of organic compounds are transferred to a menaquinone pool via a NADH dehydrogenase located in the inner membrane. Depending on the redox potential of the final electron acceptor, the electrons are transferred from the menaquinone pool to inner-membrane associated cytochromes ImcH, CbcL or CbcBA: ImcH is recruited when the final electron acceptor has reduction potential values above −100 mV (vs. standard hydrogen electrode, SHE), CbcL with values between −100 and − 210 mV, and CbcBA operates below −210 mV. The electrons are then transferred to periplasmic cytochromes (mainly to the cytochrome family PpcA) and from these to porin-cytochrome trans-outer membrane complexes [ExtABCD, ExtEFG, ExtHIJKL, Om(abc)B and Om(abc)C], which then convey the electrons to the extracellular electron acceptors. In addition, *G. sulfurreducens* is known to establish direct electrical contacts with extracellular electron acceptors via electrically conductive filaments. However, the exact mechanism of electron transport and the proteins’ nature is not yet clearly understood, with evidence on both sides: some studies identified the filaments as an assembly of repeating subunits of PilA (gene annotation number GSU1496), a type-IV pilin monomer, in which the aromatic amino acids provide a conduit for electron transfer (Reguera et al., 2005; Lovley, 2022); other studies identified that outer membrane *c*-type cytochromes OmcS, OmcZ and OmcE polymerize and transfer electrons through the stacked hemes in their inner core (Wang et al., 2019, 2022; Gu et al., 2023). The ET network of the current consuming mode is far less well-understood, but it is hypothesized that it shares a similar organization with the network used in the current producing mode, having redox partners poised at the outer membrane, periplasm, and inner membrane. However, comparative studies in current-consuming *versus* current-producing *G. sulfurreducens’* biofilms showed that the abundance of the gene transcripts is considerably distinct, and hence specific proteins are recruited for each electron transfer pathway (Strycharz et al., 2011). For example, while the gene encoding for cytochrome OmcZ, essential for current-producing pathway, had much lower abundance in current-consuming biofilms, genes encoding specific electron transfer proteins, such as periplasmic monoheme cytochromes PccH and GSU2515, show the largest transcript abundance in current-consuming biofilms (Strycharz et al., 2011). In addition, *G. sulfurreducens* biofilms in current-consuming mode have been observed to accumulate iron oxide species in their outer membrane, possibly for facilitating the ET process in this direction (Heidary et al., 2020). This metabolic versatility has permitted *Geobacter*’s application in the fields of bioremediation, for the removal of toxic compounds, such as uranium, from contaminated waters (Lovley and Phillips, 1992; Anderson et al., 2003); bioenergy, for the generation of electric energy in microbial fuel cells (Santoro et al., 2017); microbial electrosynthesis, in which electrogenic bacteria provide reductive power for the production of industrially relevant compounds such as alcohols, carboxylic acids or fuels (Gong et al., 2020).

The bacteria from the genus *Geobacter* were initially classified as strict anaerobes (Caccavo et al., 1994). However, the complete genome sequence of *G. sulfurreducens* revealed the presence of proteins typically involved in aerobic respiration and protection against oxidative stress such as: superoxide dismutase (GSU1158, GSU0720), cytochrome *c* peroxidase (GSU2813, GSU0466), catalase (GSU2100), peroxiredoxins (GSU0066, GSU0352, GSU0893, GSU3246, and GSU3447), rubrerythrins (GSU2612, GSU2814), and hydrogenases Hya (encoded by GSU0120 to GSU0123) and Hyb (encoded by GSU0782 to GSU0785) (Methé et al., 2003; Coppi et al., 2004; Tremblay and Lovley, 2012). Subsequent studies on *G. sulfurreducens* supported the genomic analysis and demonstrated that it tolerates oxygen exposure up to 24 h and can utilize this molecule as electron acceptor under microaerobic conditions (10% v/v of oxygen; Lin et al., 2004; Engel et al., 2020). This characteristic brings competitive advantage to *G. sulfurreducens* as in the water sediments near the oxic-anoxic interface, Fe(II) produced from microbial reduction can be regenerated to Fe(III) and endow continuous extracellular electron transfer processes (Lin et al., 2004). Furthermore, this offers an opportunity for the development of oxygen-resistant *G. sulfurreducens* strains that are more robust for biotechnological applications (Tremblay and Lovley, 2012; Speers and Reguera, 2021). Studies carried out for the *G. sulfurreducens* bacterium under several oxygen conditions showed two different behaviors: with low oxygen concentration (1% oxygen provided to *G. sulfurreducens* culture), the bacterium overexpresses 11 type IV pilus genes (GSU2029 to GSU2039) that are hypothesized to be involved in the motility mechanism of *G. sulfurreducens*, endowing it to flee from the oxygen- contaminated area; in high oxygen concentration environments (5% oxygen provided to *G. sulfurreducens* culture), the bacterium downregulates the expression of the type IV pilus genes and instead overexpresses genes involved in cell encapsulation and biofilm production to decrease oxygen exposure (Engel et al., 2020). These pathways were observed in a genetic study that disrupted some of the important genes identified in oxidative stress response, including the *macA* peroxidase, and observed the mutated strains’ response to oxygen (Speers and Reguera, 2021).

To date, in *G. sulfurreducens*, two NiFe periplasmic hydrogenases Hya and Hyb were shown to play a role in oxidative stress protection (Tremblay and Lovley, 2012), and two diheme proteins with homology to cytochrome *c* peroxidases were characterized: CcpA (GSU2813; Hoffmann et al., 2009; Ellis et al., 2011) and MacA (GSU0466; Butler et al., 2004; Seidel et al., 2012). Proteomic studies have shown that MacA is relevant in the reduction pathways of Fe(III) citrate (Butler et al., 2004), Fe(III) oxide (Aklujkar et al., 2013), U(VI) (Shelobolina et al., 2007), and that it may regulate the expression of OmcB, since this protein was not expressed in a *macA* deletion strain (Kim and Lovley, 2008). This latter aspect might suggest an indirect role of MacA in iron reduction (Kim and Lovley, 2008). Regarding its role in oxidative stress, MacA is able to reduce hydrogen peroxide (Seidel et al., 2012), and a *G. sulfurreducens* strain in which this gene was disrupted displayed enhanced biofilm formation and absence of oxygen tolerance, characteristic of oxidative stress susceptibility (Speers and Reguera, 2021). *In vitro*, MacA was also shown to interact and exchange electrons with PpcA (Seidel et al., 2012; Dantas et al., 2017), one of the most abundant periplasmic cytochromes.

MacA is a 35 kDa membrane-associated diheme cytochrome peroxidase located in the periplasm (Butler et al., 2004). The two hemes in this protein are functionally distinct: the His-Met axially coordinated high-potential (HP) heme receives electrons from putative electron donors and, upon reduction, triggers conformational changes that detach the distal histidine of the low-potential (LP) heme, endowing this heme with peroxidase activity (Seidel et al., 2012). The five members of the periplasmic family of triheme cytochromes (PpcA-E) are responsible for mediating ET in the periplasm (Dantas et al., 2015). Each member of the family has approximately 9.5 kDa of molecular weight and three low-spin hemes coordinated by two histidines (Dantas et al., 2015). Despite the high sequence identity, the individual heme redox properties of the five cytochromes are different (Morgado et al., 2010).

Proteomic and gene knockout studies in *G. sulfurreducens* revealed different expression levels for PpcA-E cytochromes depending on the final electron acceptor [Fe(III) citrate (Ding et al., 2008), Fe(III) oxides (Ding et al., 2008), Mn(IV) oxides (Aklujkar et al., 2013)]. Even though different expression levels were detected, a very recent study suggested that the absence of a particular triheme periplasmic cytochrome could be compensated by the overexpression of another member of the family, highlighting the versatility of these proteins (Choi et al., 2022). Furthermore, *G. sulfurreducens* strains for which each one or all triheme periplasmic cytochromes were knocked out showed more susceptibility to hydrogen peroxide, suggesting also a possible role of these proteins in radical detoxification mechanisms (Choi et al., 2022).

In this work, we directly probed ET reactions between the peroxidase MacA and the PpcA-E cytochrome family with the aim of determining the most relevant interacting pairs and elucidating the detoxification pathways involving MacA.

## 2. Materials and methods

### 2.1. Protein expression and purification

The five triheme cytochromes (PpcA-E) and MacA were overexpressed in *E. coli* BL21(DE3)::pEC86, in which the pEC86 plasmid contains the cytochrome maturation cluster *ccmABCDEFGH* for aerobic overexpression of *c*-type cytochromes (Arslan et al., 1998), and purified according to the protocols previously described (Londer et al., 2002; Morgado et al., 2007; Pokkuluri et al., 2010; Seidel et al., 2012) in an ÄKTA Prime Plus Chromatography System (GE Healthcare).

Briefly, for the triheme cytochromes, the *E. coli* BL21(DE3)::pEC86 cells were transformed with the plasmid pCK32 (encoding for each PpcA-E mature cytochrome) and grown in 2xYT media supplemented with chloramphenicol (34 μg/mL) and ampicillin (100 μg/mL) at 30°C, 200 rpm. Upon reaching an OD_600nm_ value of ∼1.5, 10 μM isopropyl β-D-thiogalactoside (IPTG) was added and the culture grew overnight at 30°C, 180 rpm. Cells were harvested by centrifugation (6,400 *xg*, 20 min, 4°C) and the cell pellet was gently resuspended in 30 mL lysis buffer [20% sucrose (Fisher scientific), 100 mM Tris–HCl (NZYTtech) pH 8.0 and 0.5 mM EDTA (Sigma), 0.5 mg/mL lysozyme (Fluka)] per liter of initial cell culture. The lysate was centrifuged at 15,000 *xg*, 20 min, 4°C and then ultracentrifuged at 150,000 *xg*, 90 min, 4°C. The clear supernatant was dialyzed twice against 10 mM Tris–HCl, pH 8.5. Purification encompassed a cation exchange chromatography step using 2 × 5 mL Bio-Scale^™^ Mini UNOsphere^™^ S Cartridges (BioRad) equilibrated with the same dialysis buffer. The protein was eluted with a 150 mL gradient of 0–300 mM NaCl. The red fractions containing PpcA-E were selected and concentrated to 1.5 mL, and then loaded onto a Superdex 75 XK16/60 molecular exclusion column (GE Healthcare) equilibrated with 100 mM sodium phosphate buffer, pH 8.0.

In the case of MacA, transformed *E. coli* cells, containing the plasmid pETSN22 that includes *macA* gene (*gsu0466*) and a N-terminal Strep-Tag(II),were grown overnight at 30°C, 200 rpm in a media supplemented with ampicillin (100 μg/mL) and chloramphenicol (20 μg/mL). No induction with IPTG was needed as the plasmid’s T7 promoter was leaky enough to obtain good protein yields. The cells were collected by centrifugation and resuspended in 2 mL per gram of cells of lysis buffer containing 20 mM Tris–HCl, pH 8.0, 250 mM NaCl. The cells were disrupted in a microfluidizer (Microfluidics) at 1,100 bar with three cycles and centrifuged a first time at 20,000 *xg*, 20 min, 4°C, and a second time at 100,000 *xg*, 1 h, 4°C. The supernatant was then purified using two steps: firstly using a streptactin superflow column (IBA), with elution of bound protein in a single step with 2.5 mM D-desthiobiotin in running buffer; secondly, by loading the eluate onto a size exclusion column (Superdex 200, 16/60, GE Healthcare) equilibrated with buffer containing 20 mM Tris–HCl, pH 8.0, 100 mM NaCl.

For the NMR studies, all proteins’ buffer was exchanged to 8 mM sodium phosphate buffer (pH 7) with sodium chloride (final ionic strength of 20 mM) followed by three cycles of lyophilization with ^2^H_2_O.

### 2.2. NMR studies

All NMR spectra were acquired in a Bruker Avance 600 MHz spectrometer at 298 K with 32 k data points, sweep width of 96.2 kHz and 1 k scans. ^1^H chemical shifts were calibrated using 2,2-dimethyl-2-silapentane-5-sulphonate as reference at 0 ppm (Wishart et al., 1995) and TOPSPIN software (Bruker Biospin) was used for spectra processing and analysis.

#### 2.2.1. MacA characterization

A lyophilized sample was resuspended in 500 μL of degassed ^2^H_2_O (final concentration 50 μM) inside an anaerobic glovebox (MBraun) with oxygen levels kept below 0.1 ppm and the NMR tube was sealed with a rubber cap. Spectra were acquired in the oxidized state (as purified), ascorbate-reduced state (1 mM sodium ascorbate in the presence of 5 μM mediator tetramethyl-1,4-phenylenediamine), and fully reduced state (50 μM sodium dithionite).

#### 2.2.2. Electron transfer reactions

The experimental procedure previously described (Morgado and Salgueiro, 2022) was followed to assess the redox reactions between PpcA-E and MacA.

NMR tubes containing each triheme cytochrome were prepared separately by resuspending lyophilized proteins in 350 μL ^2^H_2_O (for a final concentration of 50 μM in the experiment). Each PpcA-E sample was reduced by first flushing out all oxygen from the NMR tube with hydrogen, and then by adding catalytic amounts of hydrogenase from *Desulfovibrio vulgaris* (Hildenborough; Morgado et al., 2008). After complete reduction, hydrogen was removed with argon and samples were taken into the anaerobic glovebox. These procedures ensure that the PpcA-E samples do not contain oxygen. For MacA’s stock solution, the lyophilized protein was resuspended in 150 μL of degassed ^2^H_2_O (for a 200 μM final concentration in the experiment). MacA’s stock solution was added to each of PpcA-E cytochromes in molar ratios (PpcA-E:MacA) 1:1, 1:2, 1:3, and 1:4. After each MacA addition, the NMR tube was sealed again and a 1D ^1^H-NMR spectrum was immediately acquired. For the control experiment, degassed ^2^H_2_O was added to a reduced PpcA-E sample instead of MacA and a 1D ^1^H-NMR spectrum was acquired to ensure no oxygen was present in the degassed ^2^H_2_O that would be used for resuspending MacA. This step is essential to ensure that there is no oxygen contamination in the MacA sample and the observed oxidation phenomena are due to the presence of MacA and not by the presence of oxygen. At the end of the experiment (PpcA-E:MacA molar ratio 1:4), the NMR tube rubber cap was removed and the sample was exposed to atmospheric oxygen, and a final 1D ^1^H-NMR spectrum was acquired.

#### 2.2.3. Biomolecular interactions between PpcB-E and MacA

To study the interacting interface regions between PpcB-E and MacA and to complement the existing data for the redox pair PpcA-MacA (Dantas et al., 2017), NMR chemical shift perturbation of the heme methyl signals was measured by comparing 1D ^1^H-NMR spectra of isolated PpcB-E or MacA in the oxidized state with those obtained for a 1:4 PpcB-E:MacA ratio in the oxidized state.

Docking of the redox pairs was performed with the HADDOCK2.4 webserver (van Zundert et al., 2016; Honorato et al., 2021) using as input the default parameters and the information on the most affected hemes from the chemical shift perturbation experiments: HP heme for MacA, heme IV for PpcB and PpcE, heme I and heme IV for PpcC, heme III and IV for PpcD. Atomic coordinates of PpcB (PDB 3BXU), PpcC (PDB 3H33), PpcD (PDB 3H4N, chain B), PpcE (PDB 3H34), MacA (PDB 4AAL, chain A) were used for the docking. The procedure was performed with MacA’s chain A to match the conditions on the previously reported docking PpcA-MacA (Dantas et al., 2017).

## 3. Results and discussion

### 3.1. NMR fingerprints of MacA’s different redox states

**Figure 1:**
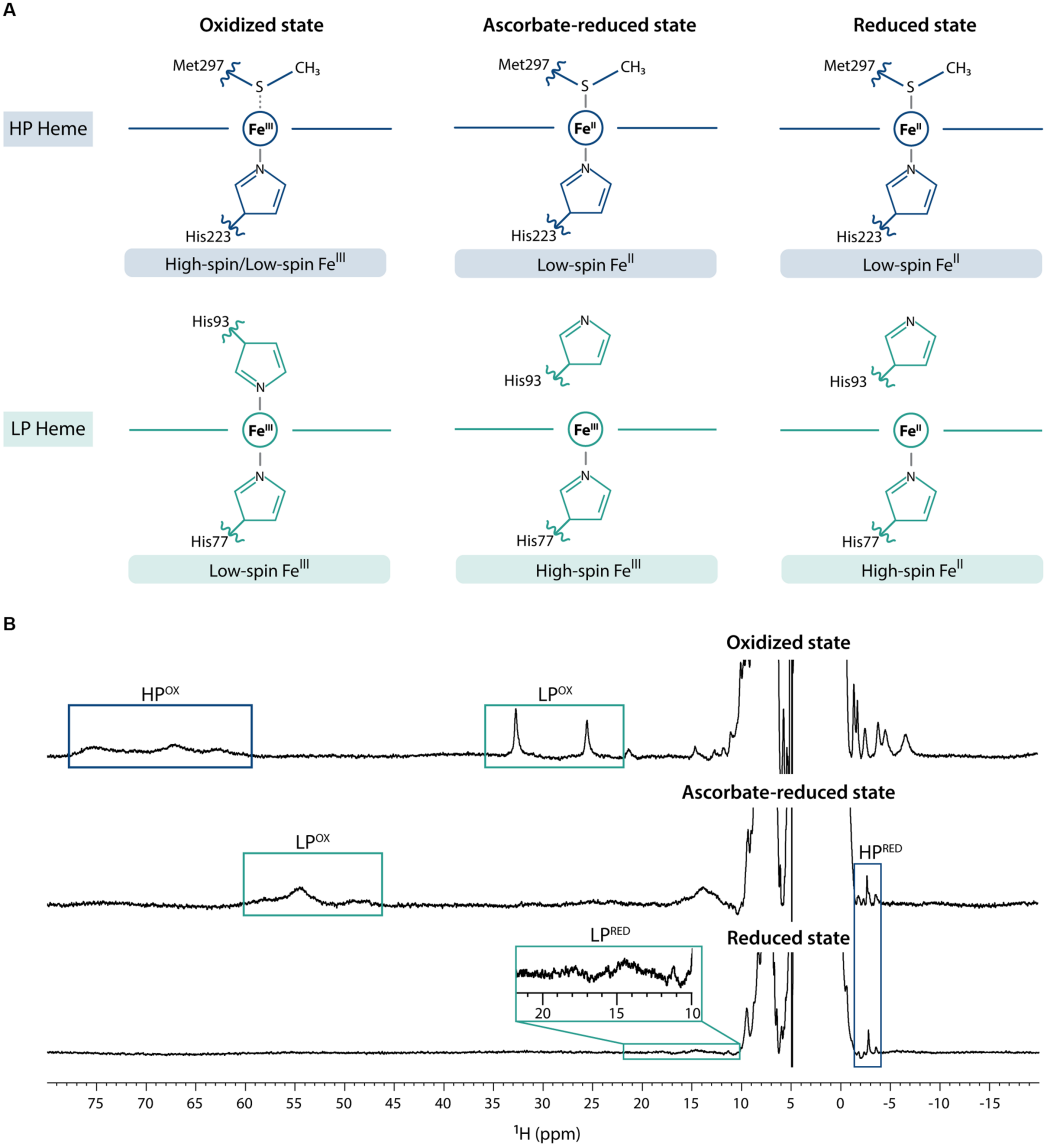
Fingerprints of MacA in the three redox states. **(A)** MacA’s heme axial coordination in the oxidized, ascorbate-reduced state, and fully reduced state with the indication of respective iron spin states, **(B)** 1D ^1^H-NMR spectra of MacA in the three different redox states (pH 7 and 298 K) with labeling of the hallmark signals. Expansion of the 10–22 ppm range in the reduced spectra highlights the high-spin signals of the LP heme.

The previous electrochemical and structural characterization of MacA (Seidel et al., 2012) showed that it can adopt three main redox states – fully oxidized, partially reduced (from hereafter referred as ascorbate-reduced), and fully reduced (by the addition of sodium dithionite) – and, as such, NMR spectra were acquired for each condition (Figure 1). In the fully oxidized state, on one hand, the HP heme (His-Met coordinated) undergoes a rapid high/low-spin equilibrium due to the weekly bound methionine (Seidel et al., 2012) and, thus, in the 1D ^1^H-NMR spectrum the heme methyl signals are broad and found in the characteristic region ranging from 60 to 80 ppm. On the other hand, those from the low-spin, LP heme (His-His coordinated) are observable in the 20–35 ppm region. Addition of ascorbate reduces the HP heme, which changes to low-spin state, causing the disappearance of 60–80 ppm signals and the appearance of a signal at −2.77 ppm, a hallmark of low-spin His-Met coordination that corresponds to the methionine’s CH_3_^ε^ group (Seidel et al., 2012). Conformational changes in MacA’s backbone associated to HP heme reduction detach the LP heme distal histidine, and this heme is now in high-spin state, which causes broadening of its signals and a shift to the 40–60 ppm region. Upon addition of sodium dithionite, both hemes are fully reduced: the HP heme maintains its low-spin state, corroborated by the observation of the axial methionine signal at −2.88 ppm, and the LP heme maintains its high-spin state with broad signals observed in the characteristic region of 10–25 ppm (Keller et al., 1972). This behavior is similar to the one observed for a cytochrome *c* peroxidase from *Pseudomonas stutzeri* (ATCC 11607) that was previously characterized (Villalaín et al., 1984). The slight variation of the chemical shift of the HP’s axial methionine CH_3_^ε^ group may be explained by local redox-linked conformation changes triggered by the LP heme (oxidized in the ascorbate-reduced state, and reduced in the fully reduced state; Villalaín et al., 1984).

### 3.2. MacA and periplasmic cytochromes can exchange electrons

In classical bacterial peroxidases the LP heme has a negative reduction potential value, whereas the HP heme has a positive one (Nóbrega and Pauleta, 2019). In the case of MacA, the redox potential value of the LP heme has been experimentally determined (−241 mV), but the value for the HP heme has not. However, since this protein is reduced by sodium ascorbate (reduction potential value of +330 mV; Njus et al., 2020) and analysis of the HP heme’s reduction potential values determined for other classical bacterial peroxidases, places MacA’s HP heme reduction potential value within the range + 320 mV (*Pseudomonas aeruginosa*; Ellfolk et al., 1983) to +450 mV (*Nitrosomonas europaea*; Arciero and Hooper, 1994). Together with the data obtained for the redox properties of the PpcA-E periplasmic triheme cytochromes (Table 1), this suggests that the latter have the necessary thermodynamic properties to reduce MacA’s HP heme (Dantas et al., 2017). The thermodynamic data is in accordance with the mechanistic description of MacA, as the HP heme is responsible for providing reducing power to the LP heme for its peroxidase activity. In fact, the lower reduction potential value of the LP heme (− 241 mV) compared to those of the PpcA-E proteins makes the reduction of the MacA’s LP heme by any triheme cytochrome very unlikely.

**Table 1:** Individual heme reduction potential values of MacA and cytochromes PpcA-E.

Protein	Midpoint reduction potential (mV)	Heme reduction potential (mV; Salgueiro et al., 2022)	Redox window (Santos et al., 2015)
MacA	–	(HP) Positive range* (LP) −241	250
PpcA	−117 (Morgado et al., 2008)	(I) −147 (III) −104 (IV) −110	285
PpcB	−137 (Morgado et al., 2008)	(I) −146 (III) −155 (IV) −119	270
PpcC	−143 (Santos et al., 2015)	ND	265
PpcD	−132 (Morgado et al., 2010)	(I) −149 (III) −96 (IV) −151	275
PpcE	−134 (Morgado et al., 2010)	(I) −154 (III) −160 (IV) −96	280

*The reduction potential value of MacA’s HP heme has not been experimentally determined. However, by comparison with homologous peroxidases, this value is in the range + 320 mV (*Pseudomonas aeruginosa*; Ellfolk et al., 1983) to + 450 mV (*Nitrosomonas europaea*; Arciero and Hooper, 1994). All values (vs. standard hydrogen electrode) refer to pH 7 except for MacA (pH 7.5). ND stands for not determined. In the periplasmic cytochromes, the hemes are numbered I, III and IV as consequence of superimposing the hemes with those of structurally homologous tetraheme cytochromes *c_3_* (Turner et al., 1997).

**Figure 2:**
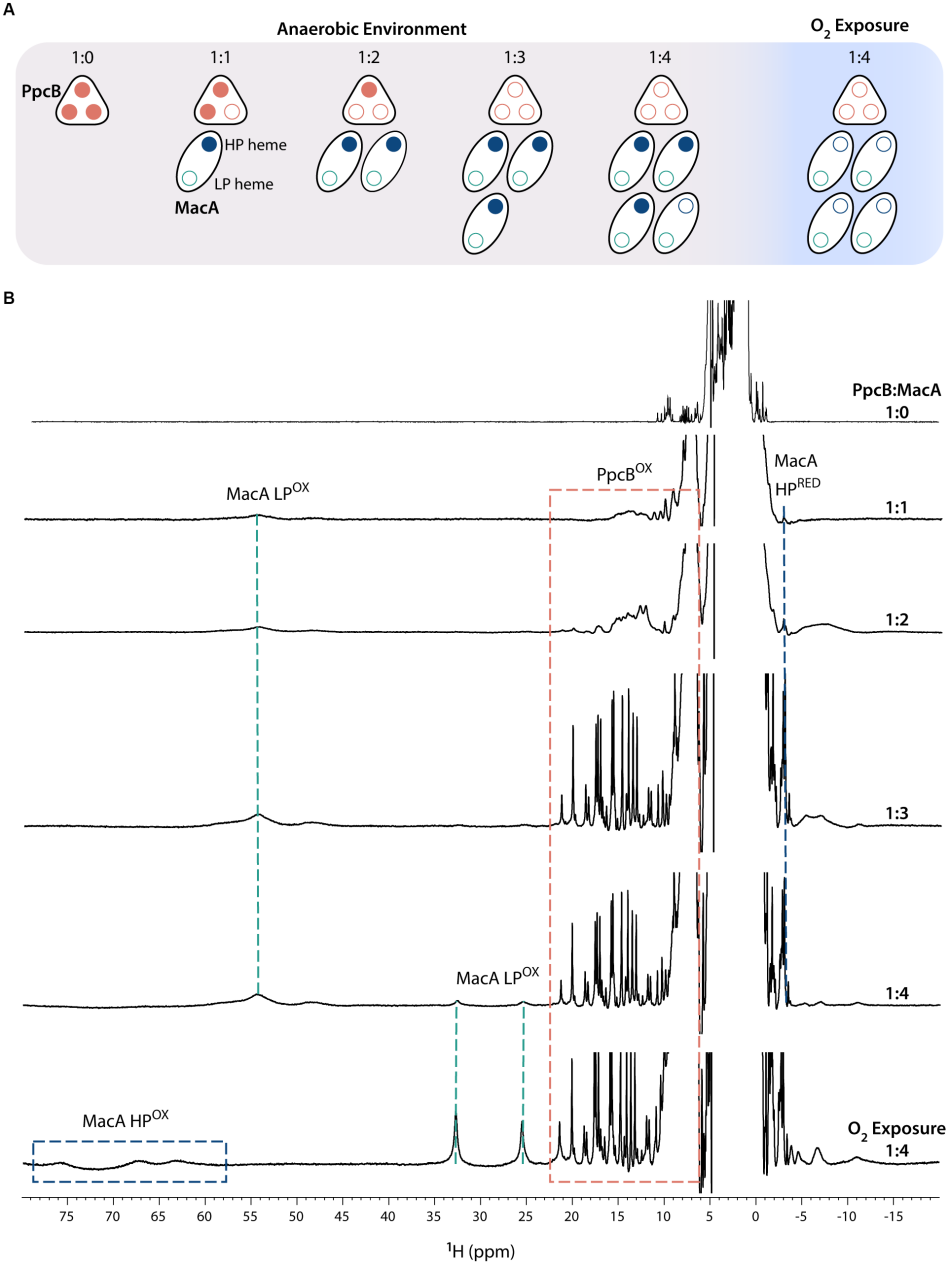
Electron transfer reaction between PpcB and MacA (pH 7 and 298 K). **(A)** Schematic representation of the experimental setup in which all additions of MacA to PpcB were done in anaerobic environment. At the end of the experiment, the proteins were exposed to atmospheric oxygen. Reduced and oxidized hemes are represented by closed and open circles, respectively. **(B)** 1D-^1^H NMR spectra acquired at the different PpcB:MacA molar ratios. The PpcB:MacA 1:0 molar ratio spectrum corresponds to the fully reduced PpcB state. The NMR spectral features of both proteins are indicated by dashed rectangles.

To test this hypothesis, ET was probed by NMR after the addition of oxidized MacA to reduced cytochromes (PpcA-E), in anaerobic conditions, at molar ratios PpcA-E:MacA 1:0, 1:1, 1:2, 1:3, 1:4 (Figure 2). The ET studies monitored by NMR rely on the different spectral features displayed by cytochromes in their different oxidation states (Morgado and Salgueiro, 2022). The signal dispersion in the oxidized state is generally wider than in the reduced state due to the paramagnetic contribution of the unpaired electron(s) to the observable chemical shifts of the heme substituents and nearby residues (Salgueiro et al., 2019). Additionally, the signal dispersion in the oxidized state is more variable, since the paramagnetic effect is determined by the specific geometry of the heme axial ligands (Turner et al., 1995; Bertini et al., 1999). In the case of MacA, the presence of a high-spin group in the oxidized state [Fe(III), S = 5/2] makes the spectral window wider (from −10 to 80 ppm) than for PpcA-E’s, whose heme groups are low-spin state [Fe(III), S = 1/2] with a typical signal dispersion from −10 to 30 ppm. In the reduced state, the spectral width is narrower and partially superimposable due to the presence of low-spin groups: PpcA-E proteins are low-spin with a spectral width from −5 to 12 ppm, and MacA possesses a low-spin (HP heme) and a high-spin (LP heme) group, thus presenting a spectral width from −4 to 25 ppm.

Figure 2 illustrates the ET reaction between PpcB and MacA. The data obtained for the other redox pairs are indicated in Supplementary Figures S1–S4. For all PpcA-E cytochromes, at 1:1 and 1:2 molar ratios, we observe the replacement of their sharp and typical reduced signals by broad signals dispersed over larger spectral widths, indicating partial oxidation of each of the triheme cytochrome. If ET occurred to both of MacA’s hemes, by the 1:2 ratio the triheme cytochromes would be fully oxidized. Instead, the observed partial oxidation suggests that only one MacA heme is participating in ET, corroborated by MacA’s spectral signature like the ascorbate-reduced state, highlighted by the presence of the HP’s reduced axial methionine CH_3_^ε^ signal and LP oxidized signals in the region 40–60 ppm. At 1:3 molar ratio, the triheme cytochromes are fully oxidized, indicating that an equimolar concentration of oxidized (triheme cytochromes) and reduced hemes (MacA’s HP heme) co-exist. Upon addition of excess MacA (1:4 ratio), while the triheme cytochromes maintain their oxidized NMR fingerprints, MacA’s LP oxidized signals start to appear, indicating that the triheme cytochromes have been depleted of reducing power and the MacA added in excess remains fully oxidized (MacA’s HP oxidized signals are not evident due to their broadness and much lower intensity than LP ones). Sample exposure to atmospheric oxygen did not affect the triheme cytochrome signals, which remained oxidized, whereas MacA’s spectral features changed to the ones indicating full oxidation, further confirming its semi-reduced state during all the anaerobic additions.

In conclusion, the ET reaction between the redox pairs is complete and only involves the HP heme. In fact, at an equimolar concentration of HP heme (PpcA-E:MacA 1:3 molar ratio) all the triheme cytochromes are fully oxidized and no intermediate oxidation state is observed. This contrasts with the redox pair CbcL:PpcA, in which the proteins maintain an intermediate oxidation state even at high molar ratios (Antunes et al., 2022). CbcL is an inner membrane associated cytochrome, with a periplasmic domain containing nine *c*-type heme groups, that plays a role in the ET to extracellular electron acceptors with low redox potential values. It was proposed that CbcL mediates the ET from the menaquinone pool to the periplasmic cytochrome PpcA. It was shown that the periplasmic domain of CbcL donates electrons to PpcA, as expected from its lower midpoint reduction potential. However, the difference of only 56 mV between the reduction potential values of the two proteins ensures an overlap of their redox windows in a way that they remain in equilibrium, acting as a reservoir of electrons to permit a constant flow whenever CbcL is loaded with electrons by the menaquinone pool and PpcA oxidized by its redox partner.

On the other hand, the results obtained for the PpcA-E:MacA complexes are in line with the physiological description of bacterial peroxidases in the literature (Nóbrega and Pauleta, 2019), as the HP heme is responsible for relaying the electrons to the LP heme with peroxidase activity. Although distinct, the midpoint and individual heme reduction potential values of PpcA-E are comparable (midpoint of, approximately, −130 mV *cf.* Table 1). Thus, considering the considerable difference between this value and that of MacA’s HP heme (in the range + 320 to +450 mV), no significant thermodynamic priorities are expected. This is indeed corroborated by our experiments, as no periplasmic cytochrome displays preferential electron transfer to MacA. Furthermore, the *in vivo* study by Choi et al. (2022) suggests that the transcription level might play a more preponderant role in oxidative stress protection: the presence of a single periplasmic cytochrome in *G. sulfurreducens* mutated strains is enough to provide oxygen stress protection, although the strains which presented less cytochrome concentration – such as the ones expressing only PpcC, PpcE, or in which all PpcA-E cytochromes were deleted – were the most susceptible ones to hydrogen peroxide.

The ability of all triheme cytochromes to reduce MacA corroborates the pivotal role of this family in providing reducing power to a great array of redox partners as observed in *G. sulfurreducens* (Choi et al., 2022). This emphasizes that any periplasmic cytochrome from the PpcA-E family may be immediately recruited for oxidative stress response, ensuring that the peroxidase MacA has all the necessary reductive power available for a prompt elimination of radical species.

### 3.3. Redox interaction interface mapping

**Figure 3:**
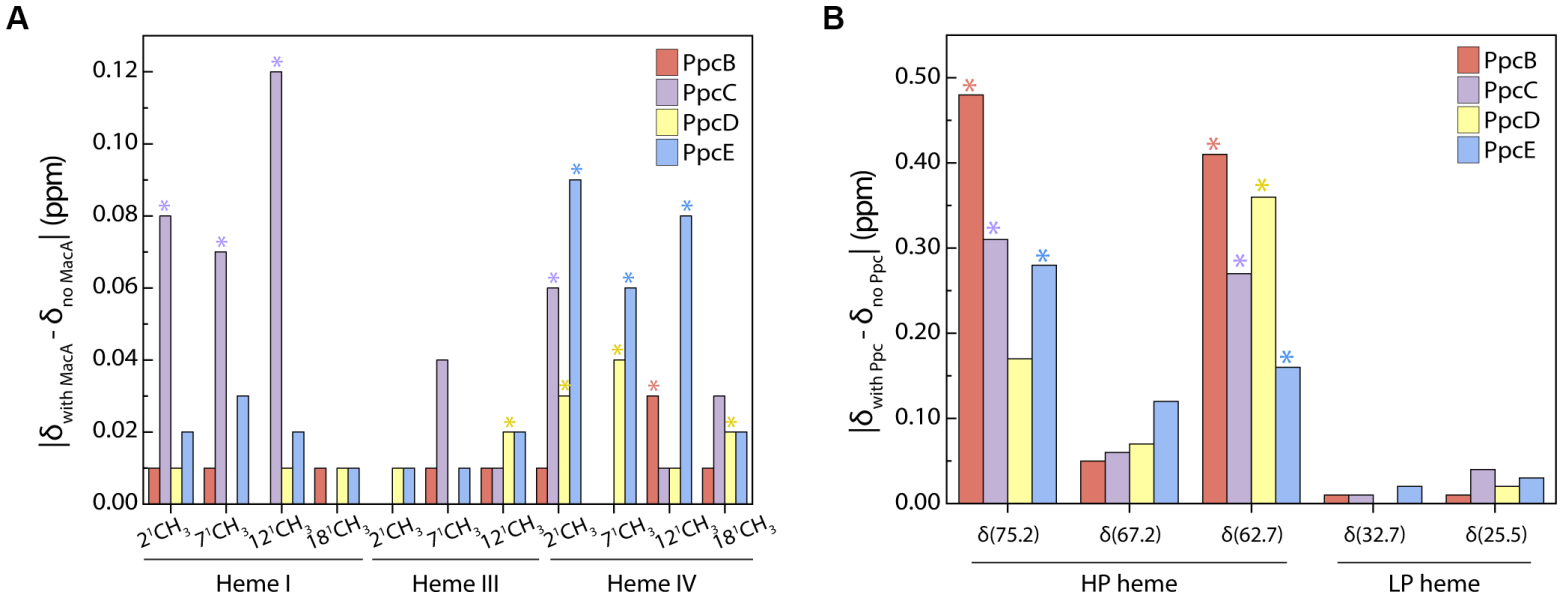
Interacting interface of triheme cytochromes and MacA. Comparison of PpcB-E’s **(A)** and MacA’s **(B)** heme substituents’ chemical shift variations in the oxidized state calculated from the data obtained in the PpcB-E:MacA 1:4 ratio. The most affected groups are indicated with an asterisk and were selected by calculating the cut-off value for each set of experiments following the procedure reported by Schumann and co-workers (Schumann et al., 2007). The cut-off values were the following: **(A)** Δδ_cut-off_ (PpcB) 0.01 ppm, Δδ_cut-off_ (PpcC) 0.05 ppm, Δδ_cut-off_ (PpcD) 0.02 ppm, Δδ_cut-off_ (PpcE) 0.04 ppm; **(B)** Δδ_cut-off_ (PpcB) 0.28 ppm, Δδ_cut-off_ (PpcC) 0.19 ppm, Δδ_cut-off_ (PpcD) 0.18 ppm, Δδ_cut-off_ (PpcE) 0.15 ppm.

Although redox interactions are characterized by their transiency, we aimed to identify whether a preferential interface existed when triheme cytochromes transfer electrons to MacA by probing the chemical shift variation of the heme methyl groups. Analysis of the most perturbed signals (Figure 3) shows that the interacting interface in the triheme cytochromes involves, in larger extent, heme IV – except for PpcC in which heme I’s methyls are the most affected ones – and MacA’s HP heme. PpcC also has an affected substituent in heme IV and PpcD has one in heme III.

**Figure 4:**
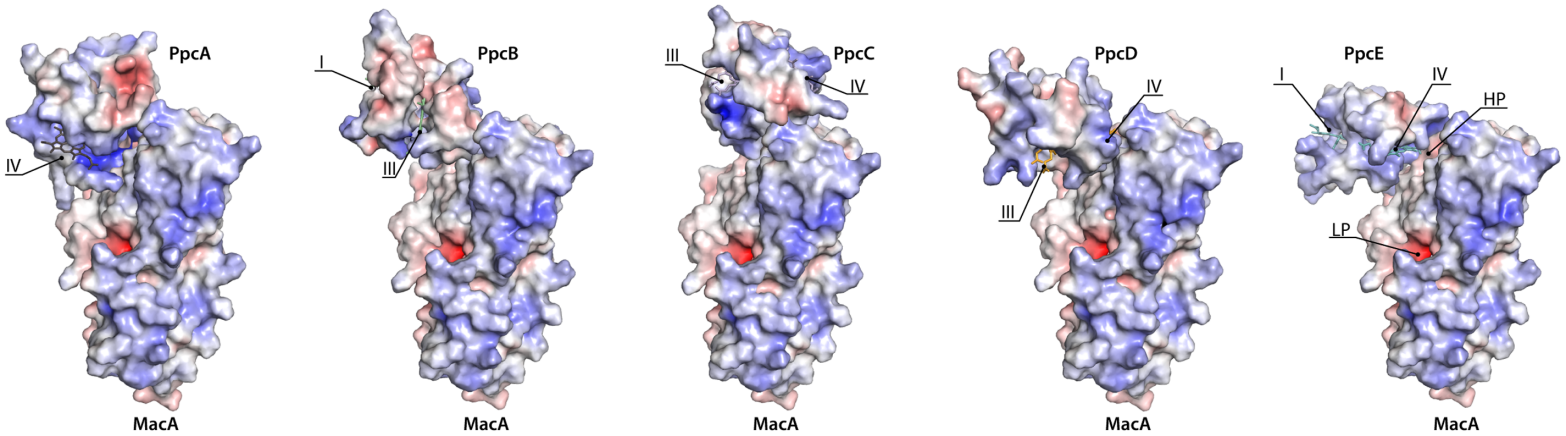
Docking of triheme cytochromes with MacA peroxidase monomer. The best docking solutions found by HADDOCK2.4 (van Zundert et al., 2016; Honorato et al., 2021) for the pairs PpcB-E with MacA are represented. Docking of PpcA with MacA is shown for the sake of completeness since it had been determined previously (Dantas et al., 2017). MacA is represented in the same orientation. The molecules’ surfaces depict the electrostatic potential, −7*k_B_T* (red) to +7*k_B_T* (blue), calculated by APBS (Dolinsky et al., 2007; Konecny et al., 2013). HP and LP stand for MacA’s HP heme and LP heme and the triheme cytochromes’ hemes are indicated by roman numbers. Structures rendered with PyMOL (Schrodinger, 2015).

Docking of the triheme cytochromes to MacA (Figure 4) showed that the interaction between the redox pairs is, most likely, driven by electrostatic charge, as the triheme cytochromes’ hemes relevant for the interaction are located within a positive electrostatic surface and MacA’s HP heme is in a negative electrostatic patch. The interaction results obtained are in accordance with a hypothesis proposed by Pokkuluri et al. (2010) that pointed out that all triheme cytochrome homologs have the highest structural similarity and positive electrostatic surface potential near heme IV, thus suggesting that ET to the redox partners occurred in this region. In the case of PpcC there is a partial positive charge surrounding heme I, as well, that may favor electrostatic attraction. However, redox interactions may be also governed by hydrophobic interactions (Nóbrega and Pauleta, 2019) and, as such, the combination of these two elements may explain the different interaction interface in this cytochrome.

**Figure 5:**
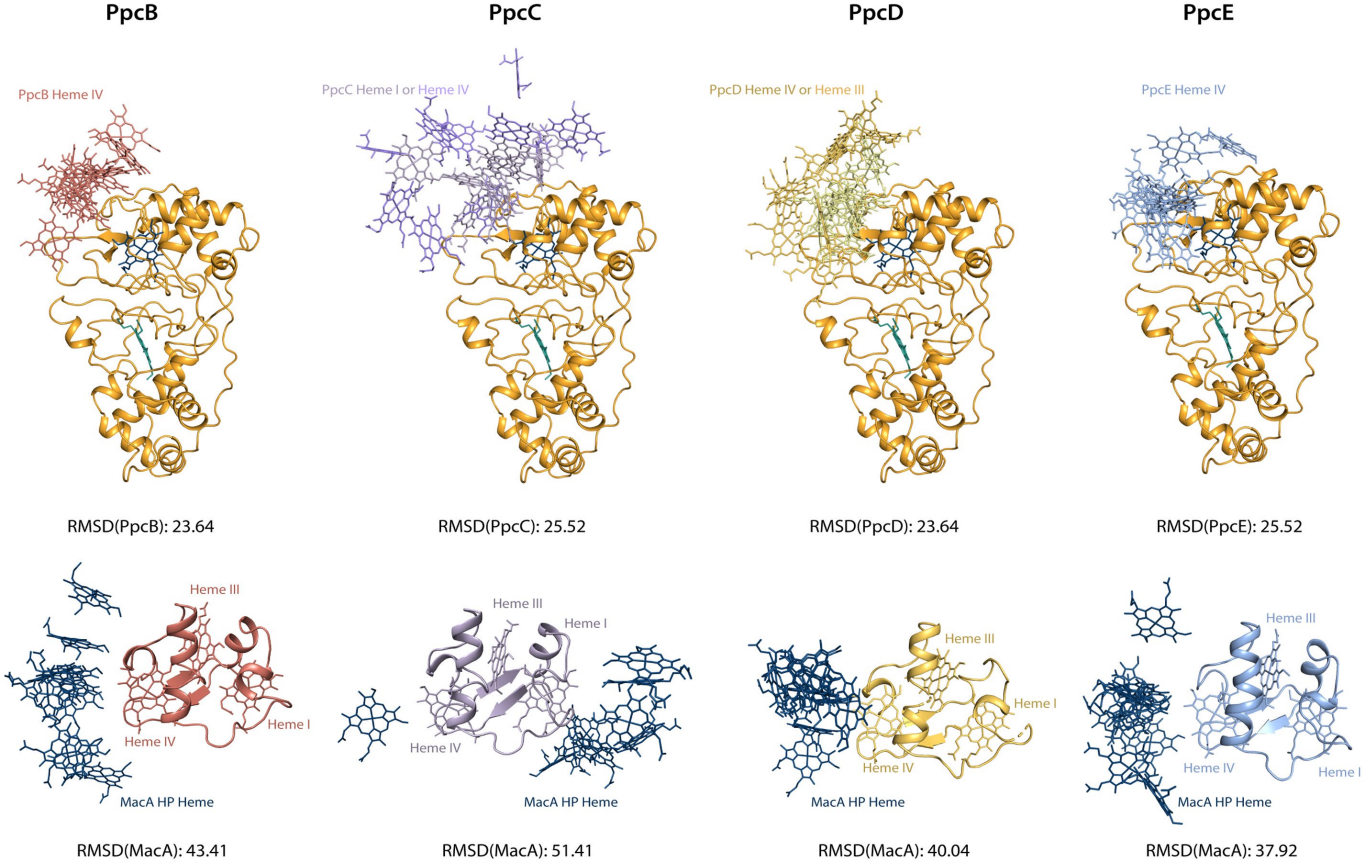
Docking solutions of triheme cytochromes and MacA peroxidase. The alignment of the complexes was made by forcing alignment of all structures to the best structure determined by HADDOCK2.4 either through MacA (top structures) or triheme cytochrome (bottom structures). The best structure of each calculated cluster is represented, as well as the RMSD value. Only the relevant interacting hemes are represented: HP heme for MacA, heme IV for PpcB and PpcE, hemes I and IV for PpcC, hemes III and IV for PpcD. Structures generated with PyMOL (Schrodinger, 2015).

The chemical shift perturbation values observed in these interactions are within the range of reported labile complexes (Worrall et al., 2003). Interestingly, the values obtained for triheme cytochromes are lower than MacA’s and this may be explained in terms of the possible orientation of the molecule in the transient complex: for MacA, the productive encounter only happens near the HP heme, thus the favorable interaction interface is much smaller than for the triheme cytochromes, as they may adopt a higher number of orientations that still allow for a productive encounter since all hemes possess a suitable reduction potential for donating an electron. To investigate this hypothesis, we aligned the structures from the best docking solutions either through MacA or the triheme cytochromes (Figure 5). Then, using Chimera (Pettersen et al., 2004) we determined the RMSD of the structure that was not aligned, i.e., the triheme cytochrome’s RMSD in the first case and MacA’s RMSD in the latter, to quantify the variability of docking positions. The results show that the RMSD values for the triheme cytochromes’ positioning on MacA are lower than the reverse situation, illustrating that the spatial solutions for a productive encounter on MacA are lower than for the triheme cytochromes.

## 4. Conclusion

Recent studies suggested that MacA and the PpcA family of periplasmic triheme cytochromes participated in the oxidative stress protection pathways in *G. sulfurreducens* (Speers and Reguera, 2021; Choi et al., 2022). In this work, direct ET from the periplasmic triheme cytochromes (PpcA-E) to MacA was probed by NMR and the results obtained showed that all the triheme cytochromes undergo complete oxidation and provide all their reducing power specifically to MacA’s HP heme. Monitorization of each redox complex interaction interface shows a higher chemical shift perturbation of MacA’s heme substituents than the triheme cytochrome ones. This indicated that, while for MacA it is necessary that the interaction occurs near the HP heme, in the case of triheme cytochromes there is a higher degree of conformations that they can adopt, thus facilitating an efficient ET. Nevertheless, this interaction is most likely driven by electrostatic complementarity of the triheme cytochrome’s positively charged patch around heme IV or heme I (in the case of PpcC), and MacA’s negatively charged patch flanking the HP heme.

Redox complexes with PpcA were also observed for cytochromes OmcF (Morgado and Salgueiro, 2022) and CbcL (Antunes et al., 2022). While the ET reaction observed in the redox pair PpcA:OmcF resembles the one observed in the present work between the PpcA-E family and MacA, in the redox pair PpcA:CbcL the two proteins remain in a semi-reduced state. This behavior illustrates the metabolic advantage of the periplasmic triheme cytochrome family, as its redox properties are versatile enough to regulate redox networks with either upstream or downstream redox partners. Finally, the results from this study provide an important contribution in the characterization of *G. sulfurreducens’* electron flow in oxidative stress conditions, highlighting the ubiquitous role of triheme periplasmic cytochromes.

## Data availability statement

The raw data supporting the conclusions of this article will be made available by the authors, without undue reservation.

## Author contributions

CS supervised the project. CS, LM, and PP designed the experiments. PP, LM, MS, and LD performed the experiments. PP, LM, and CS performed the data analysis. PP, LM, CS, and OE wrote the manuscript. All authors contributed to the article and approved the submitted version.
