# Supplementary material for: Exploring oxidative stress pathways in Geobacter sulfurreducens: the redox network between MacA peroxidase and triheme periplasmic cytochromes
Source: Front Microbiol. 2023 Oct 4;14:1253114. doi: 10.3389/fmicb.2023.1253114 (PMC10582990; doi:10.3389/fmicb.2023.1253114)
Supplement: Supplementary file 1 [file Image_1.PDF]

Supplementary Material

Exploring oxidative stress pathways in *G. sulfurreducens*: the redox network between MacA peroxidase and triheme periplasmic cytochromes

Pilar C. Portela[1,2], Leonor Morgado[1,2], Marta A. Silva[1,2], Lukas Denkhaus[3], Oliver Einsle[3], Carlos A. Salgueiro[1,2,*]

[1]Associate Laboratory i4HB – Institute for Health and Bioeconomy, NOVA School of Science and Technology, Universidade NOVA de Lisboa, 2829-516 Caparica, Portugal

[2]UCIBIO – Applied Molecular Biosciences Unit, Department of Chemistry, NOVA School of Science and Technology, Universidade NOVA de Lisboa, 2829-516 Caparica, Portugal

[3]Institut für Biochemie, Albert-Ludwigs-Universität, Freiburg, Germany

*** Correspondence:**
Carlos A. Salgueiro
csalgueiro@fct.unl.pt

1 Supplementary Figures

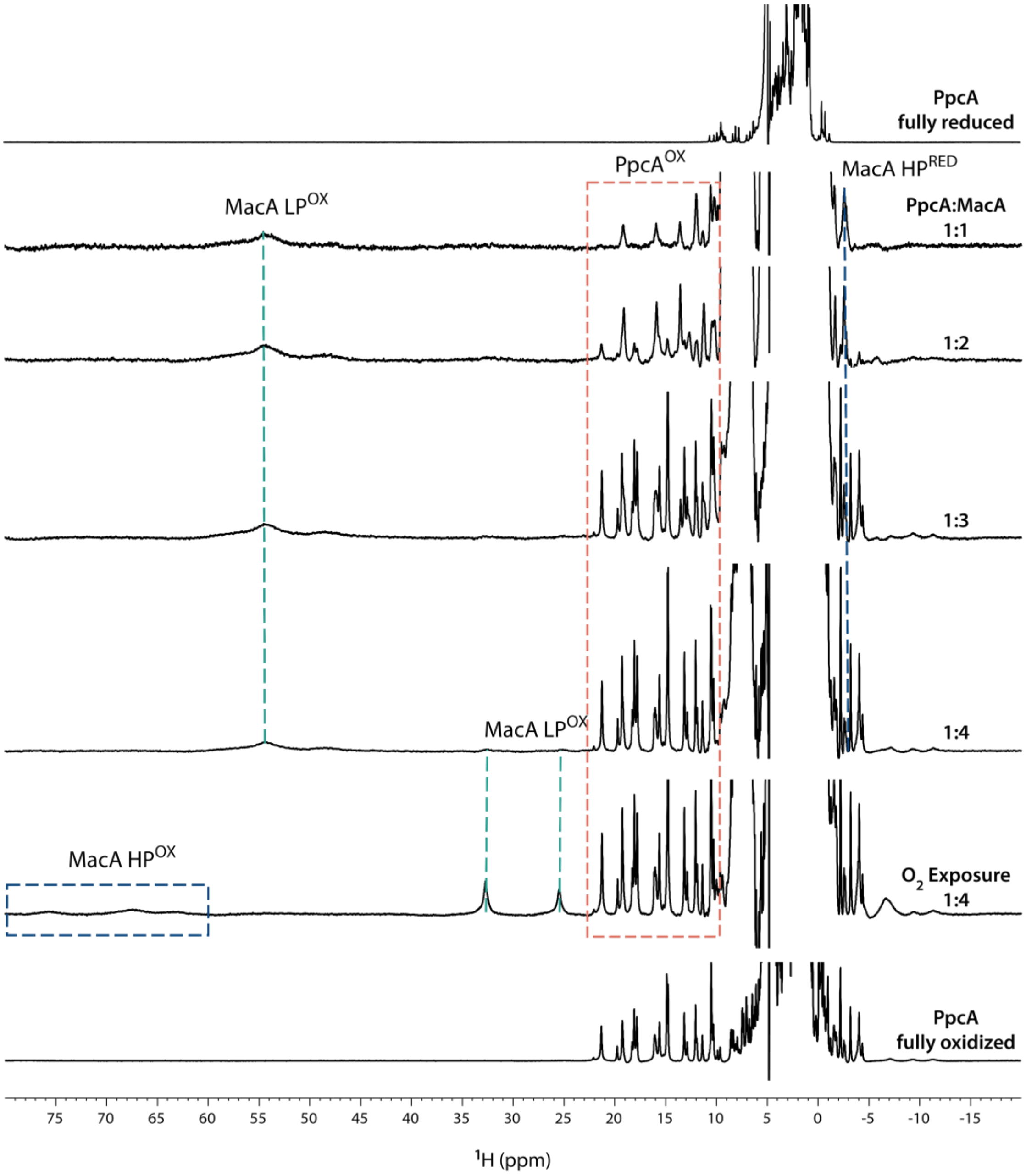

Figure S1 – Electron transfer reaction between PpcA and MacA. 1D- ^{1}H NMR spectra acquired at the different PpcA:MacA molar ratios. The fully reduced PpcA spectrum corresponds to the PpcA:MacA 1:0 molar ratio and the fully oxidized spectrum of PpcA is also included for comparison. At the end of the experiment, the proteins were exposed to atmospheric oxygen. The NMR spectral features of both proteins are indicated in the figure by dashed rectangles, with exception of MacA's HP oxidized signals in the PpcA:MacA 1:4 molar ratio spectrum due to their broadness and less intensity than MacA's LP oxidized signals

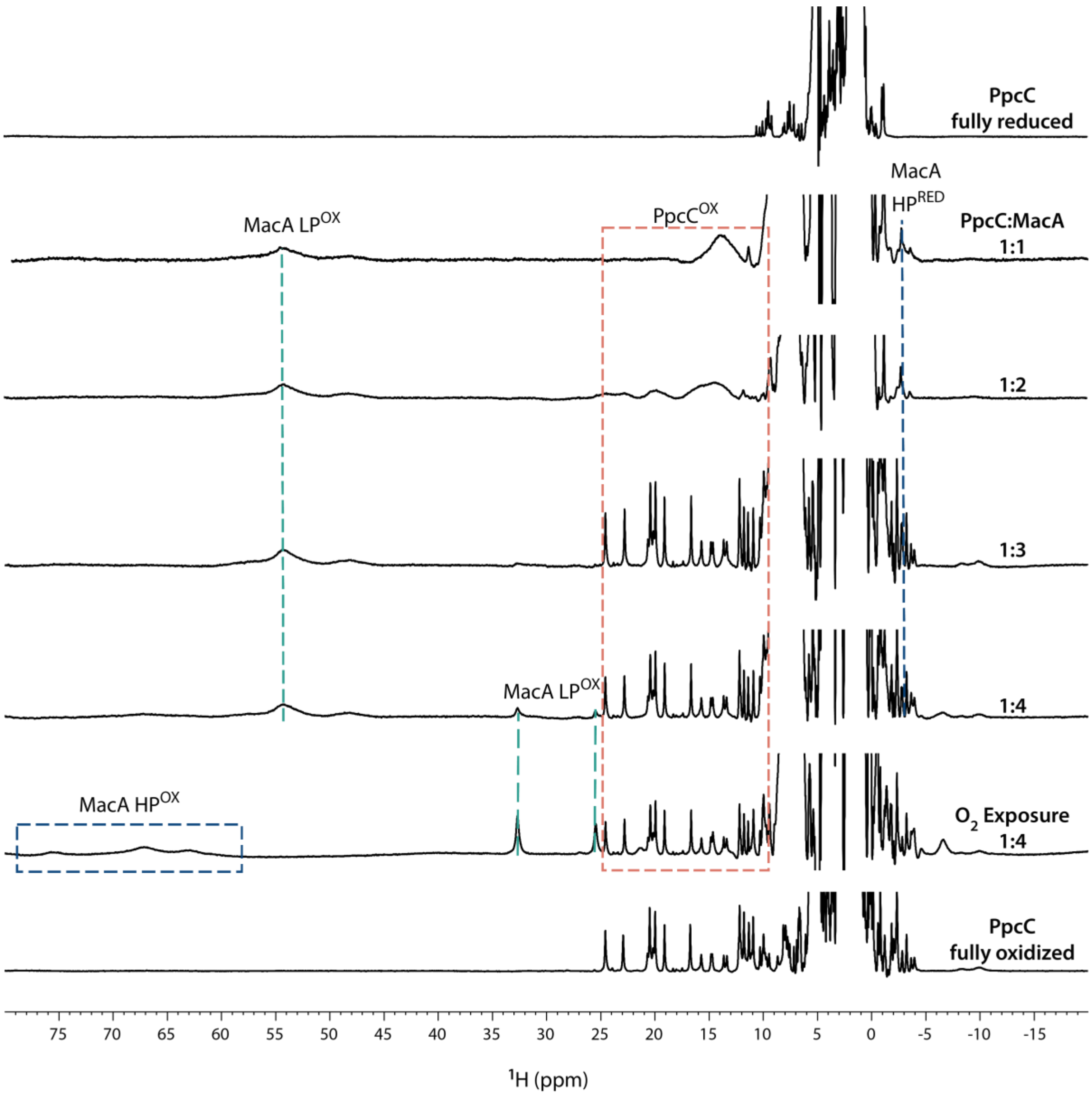

Figure S2 – Electron transfer reaction between PpcC and MacA. 1D- ^{1}H NMR spectra acquired at the different PpcC:MacA molar ratios. The fully reduced PpcC spectrum corresponds to the PpcC:MacA 1:0 molar ratio and the fully oxidized spectrum of PpcC is also included for comparison. At the end of the experiment, the proteins were exposed to atmospheric oxygen. The NMR spectral features of both proteins are indicated in the figure by dashed rectangles, with exception of MacA's HP oxidized signals in the PpcC:MacA 1:4 molar ratio spectrum due to their broadness and less intensity than MacA's LP oxidized signals.

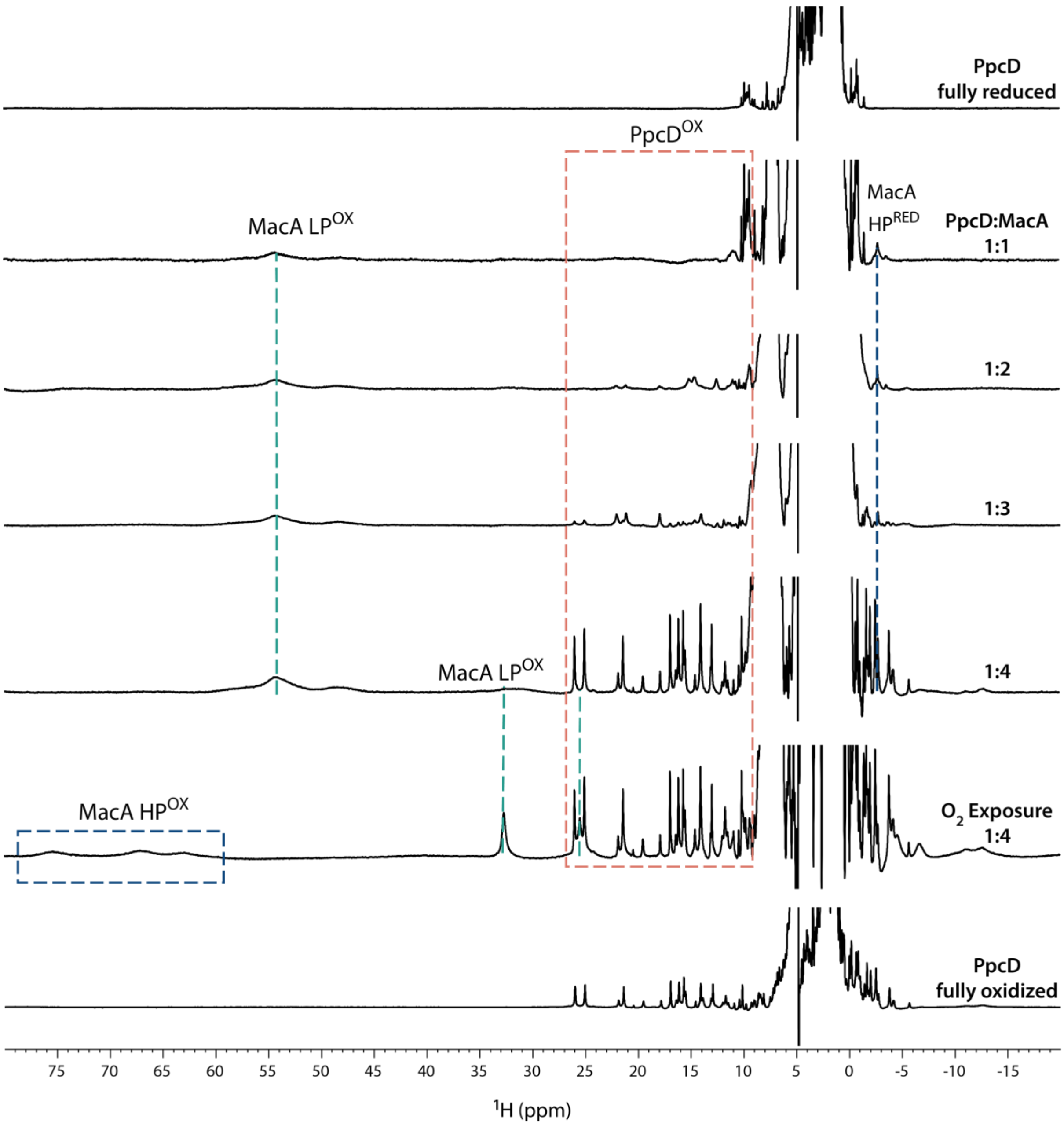

Figure S3 – Electron transfer reaction between PpcD and MacA. 1D- ^{1}H NMR spectra acquired at the different PpcD:MacA molar ratios. The fully reduced PpcD spectrum corresponds to the PpcD:MacA 1:0 molar ratio and the fully oxidized spectrum of PpcD is also included for comparison. At the end of the experiment, the proteins were exposed to atmospheric oxygen. The NMR spectral features of both proteins are indicated in the figure by dashed rectangles. In the PpcD:MacA 1:4 molar ratio spectrum, both MacA's HP and LP oxidized signals are broad since there is not a high amount of oxidized MacA in the sample.

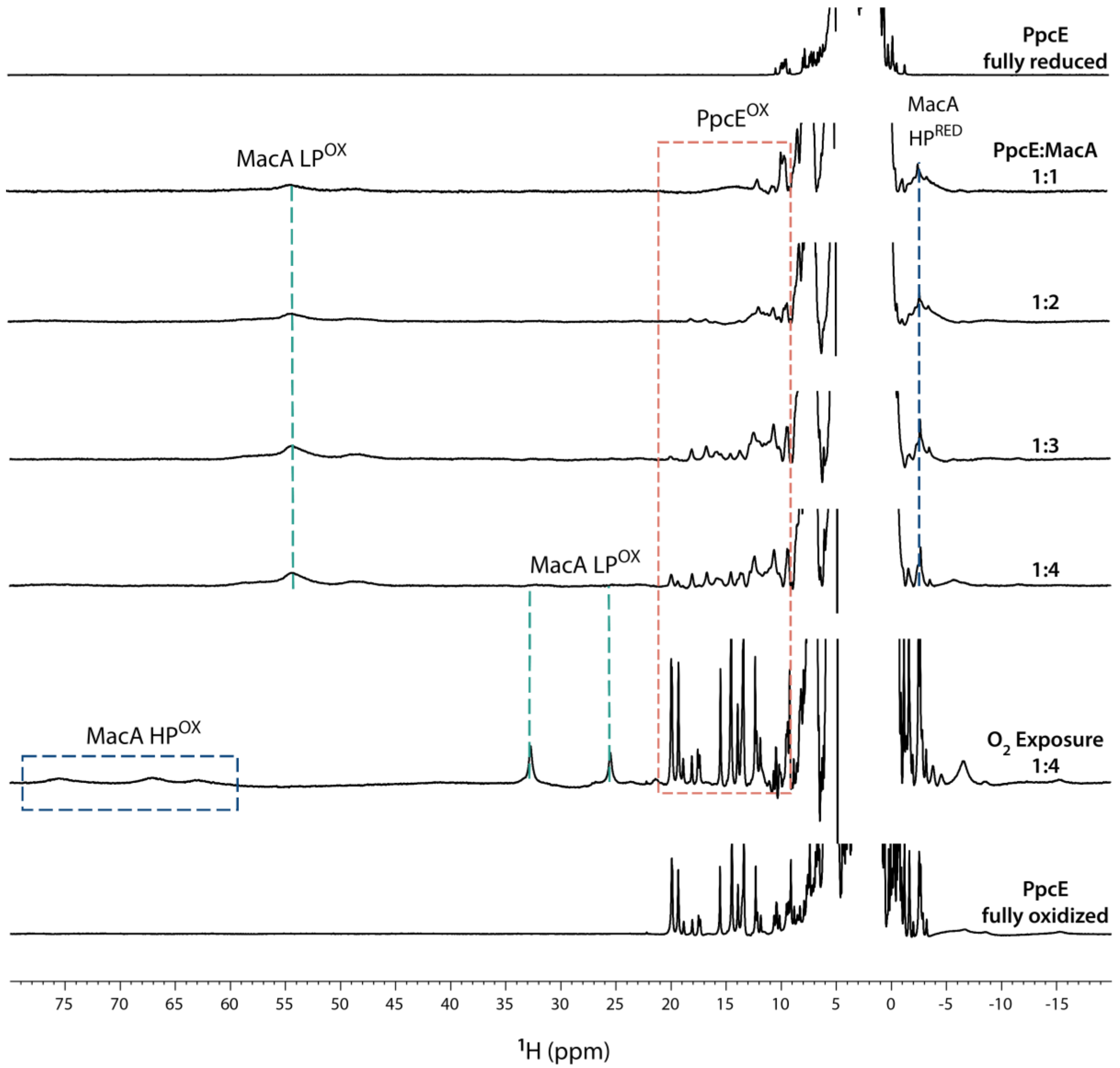

Figure S4 – Electron transfer reaction between PpcE and MacA. 1D- ^{1}H NMR spectra acquired at the different PpcE:MacA molar ratios. The fully reduced PpcE spectrum corresponds to the PpcE:MacA 1:0 molar ratio and the fully oxidized spectrum of PpcE is also included for comparison. At the end of the experiment, the proteins were exposed to atmospheric oxygen. In the PpcE:MacA 1:4 molar ratio spectrum, both MacA's HP and LP oxidized signals are broad since there is not a high amount of oxidized MacA in the sample.
